# Tumor T_1_ Relaxation Time for Assessing Response to Bevacizumab Anti-Angiogenic Therapy in a Mouse Ovarian Cancer Model

**DOI:** 10.1371/journal.pone.0131095

**Published:** 2015-06-22

**Authors:** Murali K. Ravoori, Masato Nishimura, Sheela P. Singh, Chunhua Lu, Lin Han, Brian P. Hobbs, Sunila Pradeep, Hyun J. Choi, James A. Bankson, Anil K. Sood, Vikas Kundra

**Affiliations:** 1 Department of Cancer Systems Imaging, U.T.- M.D. Anderson Cancer Center, Houston, Texas, United States of America; 2 Department of Obstetrics and Gynecology, The University of Tokushima Graduate School, Tokushima, Japan; 3 Department of Gynecologic Oncology, U.T.- M.D. Anderson Cancer Center, Houston, Texas, United States of America; 4 Department of Biostatistics, U.T.- M.D. Anderson Cancer Center, Houston, Texas, United States of America; 5 Department of Imaging Physics, U.T.- M.D. Anderson Cancer Center, Houston, Texas, United States of America; 6 Department of Cancer Biology, U.T.- M.D. Anderson Cancer Center, Houston, Texas, United States of America; 7 Center for RNA Interference and Non-Coding RNA, U.T.- M.D. Anderson Cancer Center, Houston, Texas, United States of America; 8 Department of Radiology, U.T.- M.D. Anderson Cancer Center, Houston, Texas, United States of America; University of Sheffield, UNITED KINGDOM

## Abstract

**Purpose:**

To assess whether T_1_ relaxation time of tumors may be used to assess response to bevacizumab anti-angiogenic therapy. Procedures: 12 female nude mice bearing subcutaneous SKOV3ip1-LC ovarian tumors were administered bevacizumab (6.25ug/g, n=6) or PBS (control, n=6) therapy twice a week for two weeks. T_1_ maps of tumors were generated before, two days, and 2 weeks after initiating therapy. Tumor weight was assessed by MR and at necropsy. Histology for microvessel density, proliferation, and apoptosis was performed.

**Results:**

Bevacizumab treatment resulted in tumor growth inhibition (p<0.04, n=6), confirming therapeutic efficacy. Tumor T_1_ relaxation times increased in bevacizumab treated mice 2 days and 2 weeks after initiating therapy (p<.05, n=6). Microvessel density decreased 59% and cell proliferation (Ki67+) decreased 50% in the bevacizumab treatment group (p<.001, n=6), but not apoptosis.

**Conclusions:**

Findings suggest that increased tumor T_1_ relaxation time is associated with response to bevacizumab therapy in ovarian cancer model and might serve as an early indicator of response.

## Introduction

Angiogenesis is the process of new blood vessel growth from pre-existing vessels and is crucial for cancer cell growth. Angiogenic vessels tend to be abnormal, for example, they have heterogeneous and disorganized structure [1] and increased permeability. The process is regulated by a balance of proangiogenic and antiangiogenic factors, which, upon the switch of tumor cells to an angiogenic phenotype, leads to tumor growth and progression. The rationale of anti-angiogenic therapy is based on the concept that normalization of tumor vasculature, pruning of abnormal vessels, and blockage of new angiogenesis causes tumor stabilization [2,3]. The vascular endothelial growth factor (VEGF) family of proteins and receptors play key roles in this process.

Bevacizumab (Genentech, Inc., South San Francisco, CA) is a humanized anti-VEGF monoclonal IgG1 antibody that inhibits the VEGF pathway. It was first approved by the U.S Food and Drug Administration (FDA) in 2004 for the treatment of advanced colorectal cancer in combination standard chemotherapy. Subsequently, it was approved for the treatment of advanced non-small cell lung cancer, kidney cancer and glioblastoma [4–12].

Ovarian cancer is the most lethal among gynecologic malignancies and therapies beyond surgery are not very effective. Therapies targeting the stroma, such as angiogenesis inhibitors, represent novel approaches. In ovarian cancer, prospective clinical trials have been conducted using Bevacizumab [13–16]. Among these, the largest single agent trial, Gynecologic Oncology Group (GOG) 170D trial, found a 21% response rate in patients with recurrent ovarian cancer; and, 40% of patients were progression free at 6-months [13]. Predicting response would aide patient selection for efficacy and avoid futile therapy and its complications. The mechanism of bevacizumab is to bind circulating VEGF, which inhibits its binding to its receptors on endothelial cells, thereby, altering their function and growth. The functional changes such as decreased capillary permeability and morphologic reduction in micro-vascular number should result in altered tissue characteristics, such as spin-lattice relaxation time constant (T_1_). Magnetic resonance imaging (MRI) provides excellent imaging resolution and contrast without ionizing radiation. It is based on the measurement of the spatial distribution of hydrogen nuclei, as weighted by relaxation processes that are based on their local and molecular environment in a magnetic field. T_1_ characterizes the rate of return of longitudinal signal to equilibrium. T_1_-weighted images are commonly viewed clinically, but are usually not obtained quantitatively. In this work, we employ quantitative T_1_ assessment. Tissues have characteristic relaxation time constants that can be altered physiologically or by disease [17–19]. Thus, therapy also has potential to alter T_1_. Using T_1_ for predicting response to angiogenic therapy is under investigation [20, 21]. Whether the T_1_ relaxation times of tumors can be used to assess response to angiogenic therapy with bevacizumab is not known for ovarian cancer. We assessed whether the T_1_-value of tumors could be used to assess response to bevacizumab anti-angiogenic therapy in a human ovarian cancer xenograft model.

## Material and Methods

### Cell culture

The ovarian cancer cell line SKOV3ip1-LC derived from ovarian adenocarcinoma was cultured in RPMI-1640 medium supplemented with 10% FBS and antibiotics (50μg/mL gentamicin sulfate, Cellglo, Medlatech, Inc. Manassas VA) at 37°C in a 5% carbon dioxide atmosphere.

### Animals

Female athymic nude mice (NCr-nu) were purchased from the National Cancer Institute, Frederick Cancer Research and Development Center (Frederick, MD) and maintained in specific pathogen-free conditions. The animals were cared for according to guidelines set forth by the American Association for Accreditation of Laboratory Animal Care and the US Public Health Service Policy on Human Care and Use of Laboratory Animals. All mouse studies were approved and supervised by the University of Texas-M.D. Anderson Institutional Animal Care and Use Committee (protocol 01-12-01331). The protocol included sacrifice of animals that are moribund or that have excess tumor burden.

1x10^6^ cells were subcutaneously injected in 12 female nude mice at the flank near the spine to mitigate motion on subsequent MR. Two weeks later, MR imaging was performed to assess tumor size and to obtain T_1_ maps pre-therapy. Subsequently, the animals were randomly divided into two groups of six each. The treatment group received intraperitoneal injection of bevacizumab (6.25ug/g in 200uL of phosphate buffered saline, PBS) twice a week for two weeks. The control group received intraperitoneal injections of PBS on the same schedule. MR imaging was also performed two days and two weeks after initiating therapy. Two weeks after the start of therapy, the mice were sacrificed, tumor weight was recorded, and tissue divided for formalin fixation or were frozen in optimal cutting temperature (OCT) media.

### Magnetic Resonance Imaging

MR imaging was performed pre-therapy then two days and two weeks after initial treatment. All MR studies were performed using a 4.7T scanner (Bruker Biospec, 47/40 USR, Bruker Biospin, Billerica, MA) with a 60-mm gradient insert and a volume resonator with a 35 mm inner diameter. Animals were anesthetized with 2% isofluorane and placed head first and prone on a positioning sled. Orthogonal 3-plane scout scans were initially acquired to confirm animal positioning. Animal placement and tumor location were confirmed using axial and coronal T2-weighted images (repetition time = 2750 ms, effective echo time (TE) = 50 ms, echo train = 8; field of view (FOV) = 4cm x 3cm, slice thickness = 1 mm, image matrix = 128 x128, number of signal averages = 2). Quantitative T_1_ assessment was performed. A fast spin-echo saturation-recovery sequence (TR = 110–10,000 ms [110ms, 200ms, 400ms, 600ms, 1,000ms, 2,000ms, 4,000ms, 6,000ms, 10,000ms]; TE = 50 ms; echo train = 8; field of view (FOV) = 4cm x 3cm; Image matrix = 128 x128; number of signal averages = 1) was used to measure the T_1_ of tumor tissue using regions of interest (ROI’s) encompassing the entire tumor. T_2_-weighted images were used to aide identification of tumor margins. Paravision version 4 was used to calculate the T_1_ relaxation values by exponentially fitting of signal as a function of TR. Manually drawn regions of interest on each T2-weighted image containing tumor were used to derive tumor weight as described previously [22]. Briefly, using Image J software (National Institutes of Health, USA), in each image containing a tumor, the periphery of the mass was traced and the area of the drawn region was calculated. The areas were then multiplied by the slice thickness plus skip to obtain the volume of tumor in each slice and these volumes added. Assuming a tissue density of 1 g/ml, to derive weight, volume in mm^3^ was multiplied by .001 g of tissue/mm^3^.

### Immunohistochemistry

Immunohistochemical staining for Ki67 and CD31 was performed. For Ki67 staining, formalin-fixed paraffin-embedded tissue samples were cut into 5-μm sections. After deparaffinization, antigen retrieval was performed by heating the slide in a steam cooker for 10 minutes in 0.2M Tris buffer, pH 9.0. CD31 was stained [23] using frozen slides. Endogenous peroxide was blocked with 3% H_2_O_2_ in methanol for 12 minutes. After protein block with normal horse and goat serum, slides were incubated with primary antibody to Ki67 (Thermo scientific, Fremont, CA) or CD31 (PECAM-1, 1:800 rat IgG, Pharmingen, San Diego, CA) in blocking solution overnight at 4°C. After washing with PBS three times, the HRP-conjugated anti-mouse secondary antibody (DAKO, Carpinteria, CA) in blocking solution was added for one hour at room temperature. Slides were stained with DAB substrate (Phoenix Biotechnologies, Huntsville, AL) and counterstained with Gils No. 3 hematoxylin (Sigma-Aldrich, St. Louis, MO). To quantify micro-vessel density (MVD), the micro-vessels within five randomly selected 0.159-mm^2^ fields at 100X were counted. A single micro-vessel was defined as a discrete cluster or at least three cells stained positive for CD31. The presence of a lumen was required for scoring as a micro-vessel. The Ki67 labelling index was determined by counting at least 1,000 tumor cells and the index was calculated as a percentage. TUNEL assay was performed as described [23].

### Statistical analysis

For comparing groups, 2 tailed t-tests were performed using spreadsheet software (Microsoft Office Excel 2003, Microsoft, Seattle, WA). Least squares linear regression was used to evaluate the extent of linear dependence between MR derived tumor weight and excised tumor weight. Repeated measures ANOVA was used to compare the extent of relative change in T_1_ relaxation time (pre-treatment to follow-up scans at 48 hours and 2 weeks) between bevacizumab and control mice. Random intercepts were used to adjust the inference for inter-mouse heterogeneity by accounting for interdependence among the three repeated intra-mouse scans. Two-sided Wald tests using the robust estimation method described by Koller and Stahel [24] were applied to fixed effects characterizing the mean difference between Bevacizumab and control in the extent of relative change in T_1_ relaxation time at each of the two follow-up scans. This test does not assume a Gaussian distribution enabling evaluation of data even with non-normal, skewed distribution. Bonferroni correction was used to control the familywise type I error rate at the 0.05 significance level among the two tests for differences in the extent of relative change at each follow-up scan, inducing a significance threshold of 0.025 for each test. ANOVA was implemented using the statistical software R (R Development Core Team, http://www.r-project.org) version 3.0 using the *robustlmm* package.

## Results

### Tumor size

**Fig 1:**
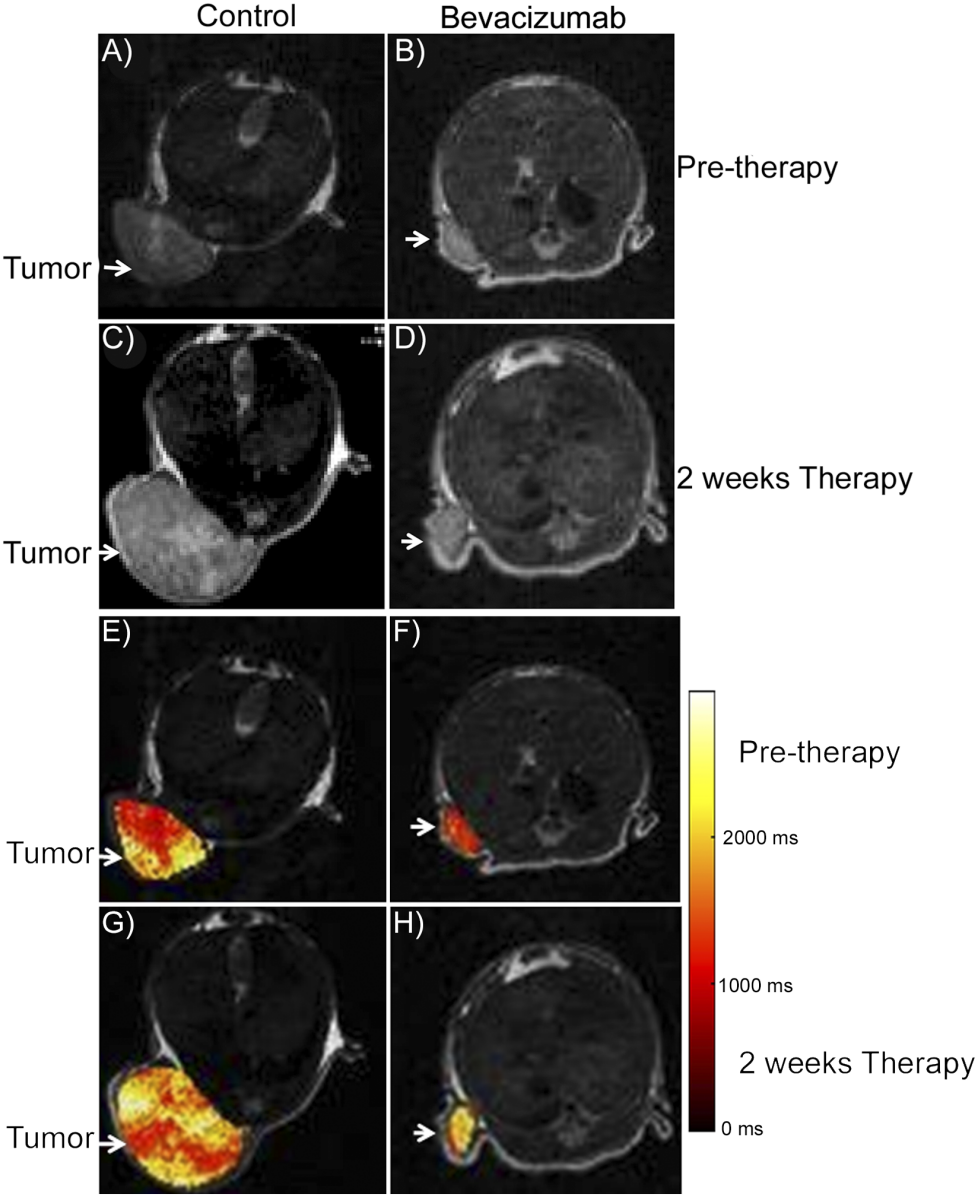
Axial T2-weighted MR imaging of mice with tumors. Representative axial MR images of mice pre-therapy (A, B) or two weeks after initiation of therapy (C, D) with vehicle (A, C) or bevacizumab (B, D). Axial T1 maps of the same tumors (E, F, G, H). Representative axial MR images of mice pre-therapy (E, F) or two weeks after initiation of therapy (G, H) with vehicle (E, G) or bevacizumab (F, H).

**Fig 2:**
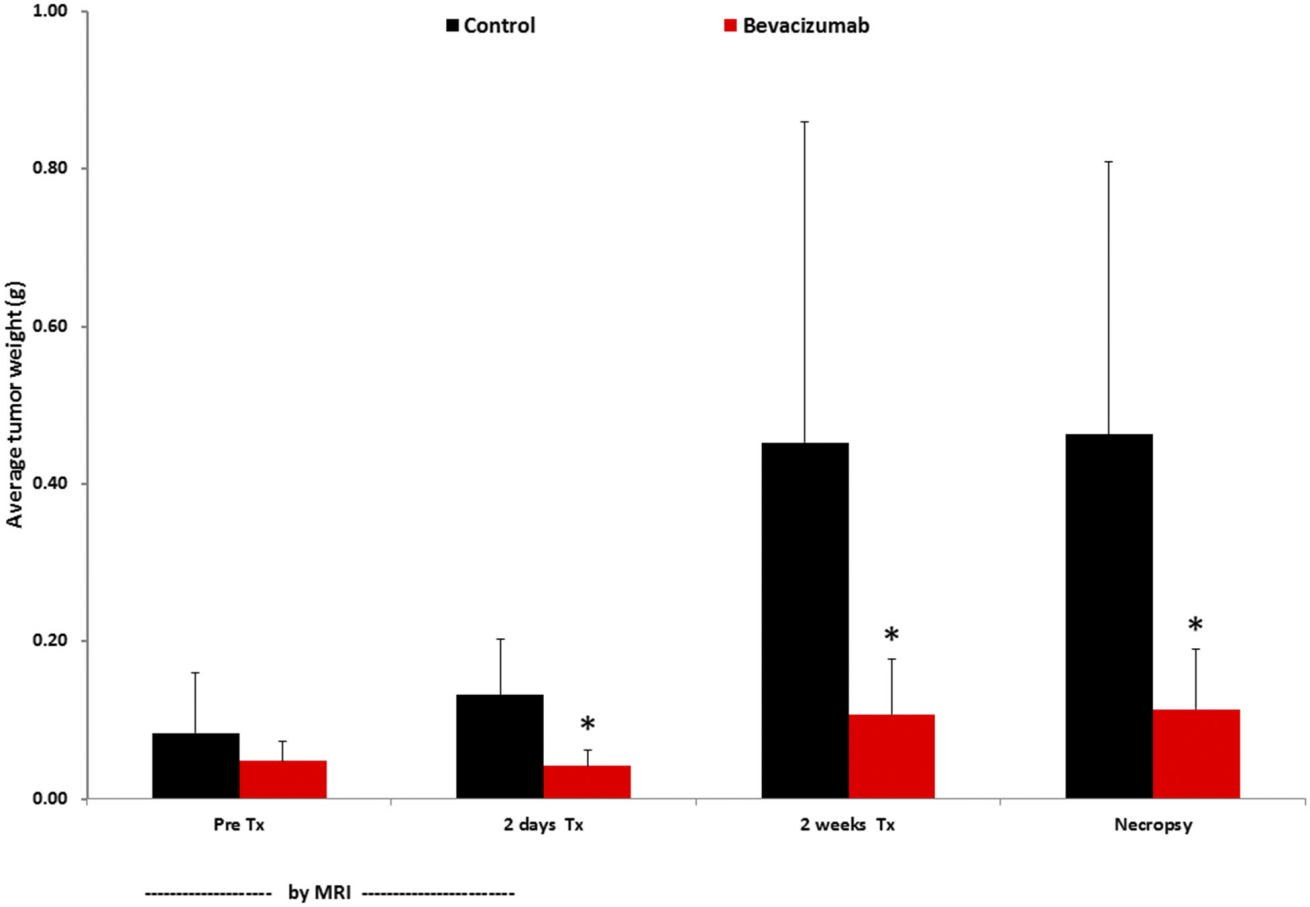
Tumor growth. Tumor weight derived from MR images before and two weeks after initiation of therapy, and, by necropsy two weeks after initiation of therapy. *, p<.05. Error bars represent standard deviation.

**Fig 3:**
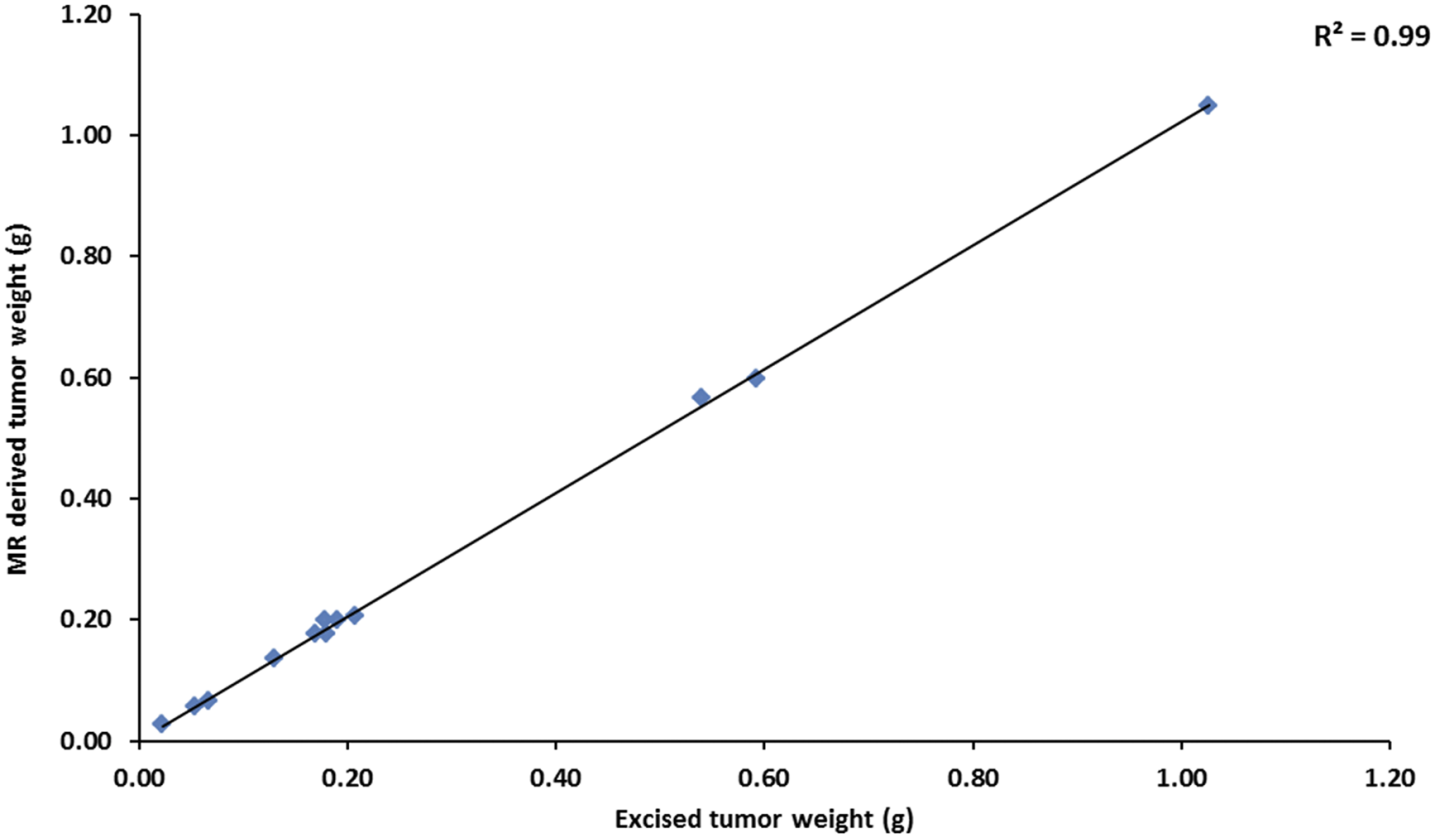
Correlation of tumor weight by MR and ex vivo.

We evaluated the effect of bevacizumab on tumor growth. Bevacizumab treatment inhibited tumor growth compared to the control as seen by MR imaging (Fig 1). Before therapy, there was no statistically significant difference in tumor weight between the two groups as measured on the MR images. Two days and two weeks post-treatment, mice treated with bevacizumab had significantly smaller tumors than control mice (p< 0.04, n = 6) by both in vivo MR measurement and ex vivo at necropsy (Fig 2). No difference was seen in tumor weights in the treatment groups pre-therapy versus 2 days or 2 weeks post initiation of bevacizumab therapy. Tumor weights derived from in vivo MR imaging correlated highly with the weights of excised tumors (r^2^ = 0.99, p<0.001, n = 12, Fig 3).

### T_1_ values

**Fig 4:**
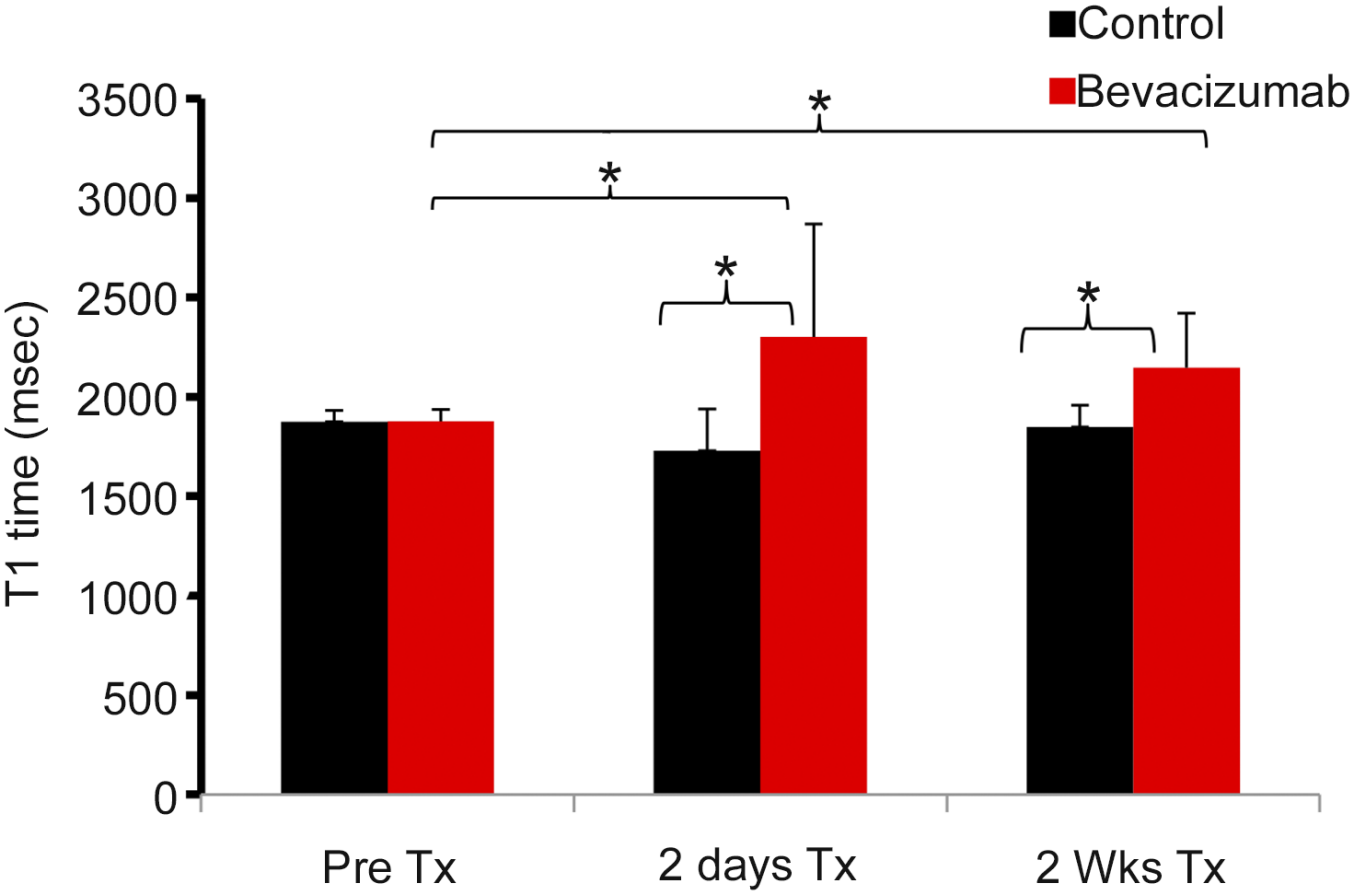
T1 values pre and post therapy. T1 values of tumors pre, 48 hour, and two weeks post therapy. *, p<.05. Error bars represent standard deviation.

The T_1_ relaxation time was significantly increased in the bevacizumab treated group compared to controls, both two days (2302±567, mean ± standard deviation, vs 1729±209) and two weeks (2146±276 vs 1847±112) after initiation of therapy (p< 0.05, n = 6, Fig 4). There was variation in the T_1_ values (S1 Fig); therefore, to adjust for potential skewness in these comparisons, two-sided Wald tests using the robust estimation method described by Koller and Stahel [24] were applied. Significant differences in the extent of T_1_ relaxation time modification were evident between bevacizumab and control cohorts at each follow-up scan after adjusting for multiplicity. Statistically, robust inference estimates that the extent of relative change in T_1_ relaxation time was 16.1% (p≤0.011) larger on average for bevacizumab treated mice when compared to control mice after 48 hours. The result maintained statistical significance after 2 weeks, where T_1_ relaxation time was increased by 14.5% (p-value≤0.021) on average for bevacizumab treated mice. Prior to treatment, T_1_ relaxation time was 1877±60 for tumors from mice that subsequently were treated with bevacizumab. In comparison, the T_1_ value did not change from pre-therapy levels (1875±57) in the control group despite increase in tumor size.

### Histology

**Fig 5:**
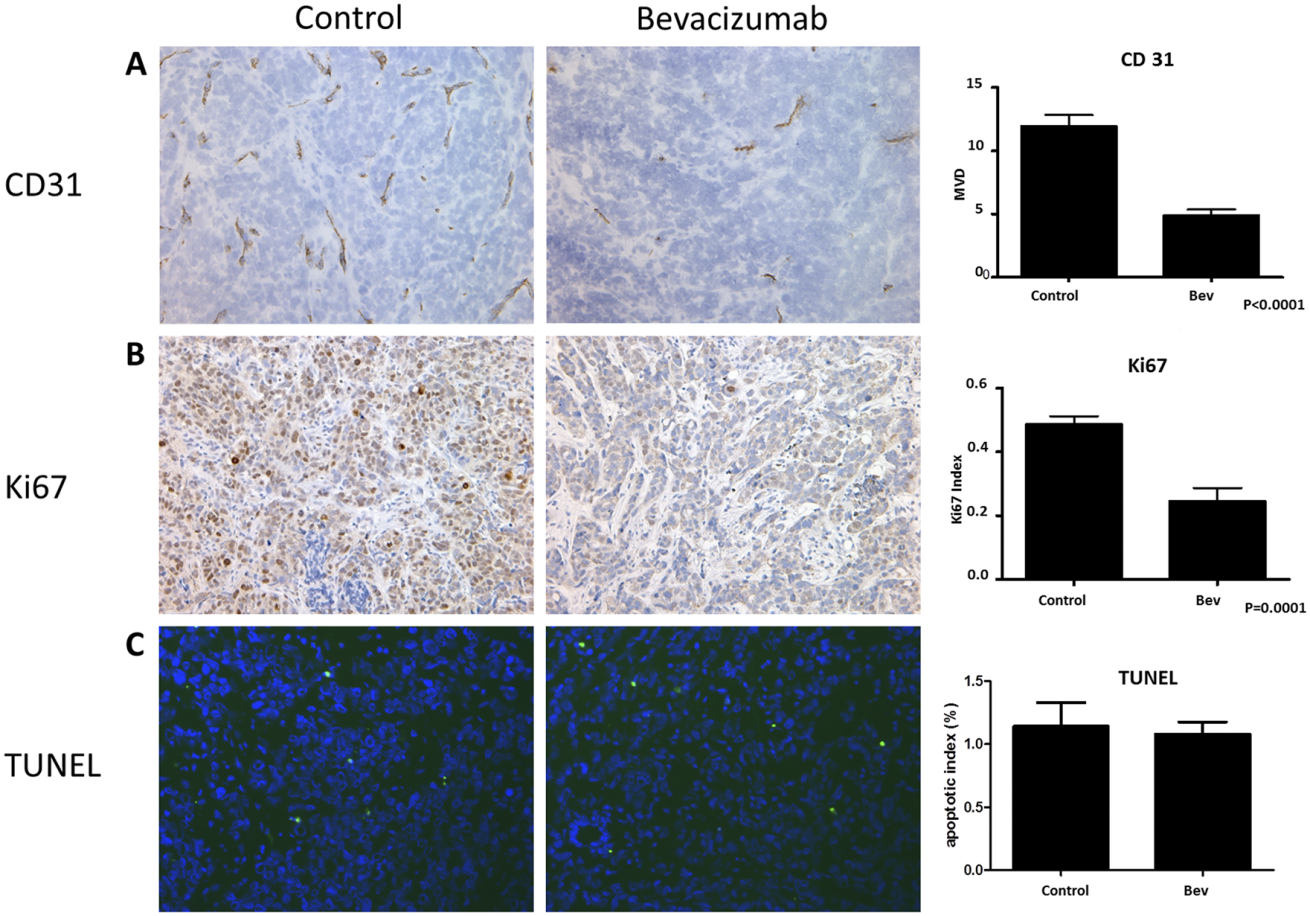
Histologic analysis of mean vessel density, proliferation, and apoptosis 2 weeks post-initiation of therapy. A) Immunohistochemical staining for CD31 (left) and microvessel density (right). B) Immunohistochemical staining for Ki67 (left) and Ki67 labelling index (right). C) TUNEL staining (left) and apoptotic index (right). Error bars represent standard deviation.

Mean vessel density, proliferation, and apoptosis were evaluated as histologic biomarkers of the effects of bevacizumab at two weeks post initiation of therapy. CD31 staining was used to mark vessels. The average mean vessel density was decreased by 59% in the bevacizumab treatment group compared to the control group (p<0.001, n = 6, Fig 5A). Ki67 was used as a marker of cellular proliferation. Ki67 labelling index was decreased by 50% in the bevacizumab treatment group (p<0.0001, Fig 5B) compared to the control group. On the other hand, apoptotic index, evaluated using TUNEL staining, was not different between the two groups (Fig 5C).

## Discussion

Anti-angiogenic therapy with bevacizumab results in treatment response in a subset of patients with ovarian cancer. Markers of response are needed to parse those patients who may benefit from therapy versus futile therapy for patients who would be at risk of complications without therapeutic benefit. Anti-angiogenic therapy alters the function and morphology of blood vessels within tumor tissue. Our data suggest that the increased T_1_ relaxation time of tumor tissue provides an early indication of response to bevacizumab anti-angiogenic therapy. This is the first report that we are aware that evaluates T_1_ relaxation time in the context of ovarian tumor anti-angiogenic therapy.

Histologic examination of tumor tissue showed decrease in microvessel density and proliferation in the bevacizumab treatment group. Tumor growth was also inhibited. These findings are consistent with altered tumor architecture and functional change in tumor growth. In contrast, the rate of apoptosis was not changed. These result are consistent with the data of Rapisarda et al. [25] who reported that microvessel density was decreased and hypoxia inducible factor-1 (HIF-1) dependent gene expression was increased after treatment of U251 (glioblastoma) tumors with bevacizumab, but apoptosis was not induced.

Abnormal blood and lymphatic vessels, including poor permeability selectivity due to high vascular permeability result in increased tumor interstitial fluid pressure (IFP) [26]. Anti-angiogenic treatment results in a decrease in microvessel density, vascular permeability and interstitial fluid pressure (IFP) [27]. In patients with rectal cancer, overall mean IFP decreased 15.0 mm Hg after treatment with bevacizumab [28]. This may affect fluid sensitive parameter T_1_ [26] since IFP is related to fluid in the extracellular-extravascular space.

Anti-angiogenesis therapy has been evaluated in a number of clinical trials using dynamic contrast enhanced MR imaging and these have commonly demonstrated decrease in the forward directional transfer coefficient, k^trans^, and initial area under the curve, IAUC, and in some studies, the unidirectional influx constant K_i_, but few have been linked to increased progression free or overall survival [29]. For bevacizumab, decreased k^trans^ has been noted [30] and although this is a complex parameter, the finding suggests decreased permeability. This appears to be a common finding and has also been suggested in several tumor models [31–33].

Thus, bevacizumab therapy has effects on several vascular parameters that effect tumor structure and behavior. This can affect the imaging characteristics of tumors. For example, in patients receiving cytotoxic therapy plus bevacizumab for 2–3 months, tumor morphology change by CT, including homogeneous low attenuation and sharp borders of colorectal liver metastases corresponded with decreased residual tumor [34]. MR has greater soft tissue contrast than CT. Most tumors involving the liver have a longer T_1_ than normal liver, thus, most metastases appear to be of lower signal on T_1_-weighted MR. Standard T_1_ weighted imaging is qualitative and can be influenced by a number of parameters such as hardware related effects, coil loading and sequence parameters, moreover, perception of change is dependent on windowing and leveling of images; in comparison, T_1_ relaxometry gives more quantitative results and can result in improved sensitivity to tissue biochemical and structural changes with pathology [35]. T_1_ mapping evaluates tissue T_1_ effects, removing contamination such as proton density, T_2_, and coil sensitivity that influence standard T_1_ weighted imaging [35].

The difference in T_1_ relaxivity of tissue enables differentiation of different tissue types. Disease of a tissue can alter T_1_ relaxation time, for example, it is increased in patients with cirrhosis compared to normal liver or chronic hepatitis [36], suggesting that the fibrotic architectural change in the liver lengthens this value. T_1_ relaxation time has been used in patients to derive water content in the brain [19] and increased T_1_ relaxation time was found to correspond with increased edema in the myocardium [37], i.e. increased fluid in the extravascular-extracellular space. Theoretically, by decreasing interstitial pressure, the amount of fluid in the extravascular-extracellular space should decrease with bevacizumab therapy resulting in decreased T_1_ relaxation time. However, bevacizumab has multiple other effects such as vessel pruning and normalization, and, as we noted, decreased tumor cell proliferation. We noted an increase in T_1_ relaxation time at two days and two weeks post-therapy, suggesting that other effects of bevacizumab on tissue parameters such as on blood flow, cellularity, architecture, and lymphatic drainage may have contributed to the increased T_1_ relaxation time that overwhelmed the theoretical decrease in T_1_ relaxation time expected from inhibiting vascular permeability alone.

T_1_ relaxation time has been evaluated in prior mouse studies in the setting of chemotherapy. McSheehy et al. evaluated several chemotherapeutics that cause shrinkage of tumors and found decrease in T_1_ relaxation time with response, including an angiogenesis inhibitor PTK/ZK on murine B16/BL6 melanoma tumors [38]. Weidensteiner et al. from the same group also found decrease in T_1_ relaxation time in response to an mTOR inhibitor in RIF-1 fibrosarcoma tumors and decrease in Ki67, but not mean vessel density [39]. Older studies have noted increase or decrease of T_1_ relaxation time with treatment [40–43]. Differences may be due to the therapeutic, animal model, and different methods of calculating T_1_ relaxation time. In addition, increased fractional tumor water content results in longer T_1_, and although correlations are not very strong, necrosis can result in shorter T_1_ thought to be due to release of complexed paramagnetic ions/proteins with development of necrosis [43,44]. We noted no difference in tumor size or T_1_ relaxation times between treated and untreated groups pre-therapy, and without therapy, the T_1_ relaxation time did not change despite enlarging tumors. With bevacizumab therapy, tumor growth was inhibited; and, in these tumors, as early as two days post therapy, T_1_ relaxation time increased. At two weeks, growth was still inhibited compared to the no therapy group and T_1_ relaxation time was still increased. Mechanistically, the increased T_1_ relaxation time corresponds with decreased proliferation and decreased mean vessel density as seen in Fig 5, suggesting that more extra-cellular extra-vascular space became available for fluid. A limitation of this paper is that extra-cellular extra-vascular space was not directly measured, however, prior reports also suggest that increased T_1_ suggests increased extracellular volume [45]. A potential limitation of this paper is use of subcutaneous tumors. Although orthotopic models are commonly used, so are subcutaneous models [46–49]. A problem for intra-abdominal imaging of the mouse by MR is that it is prone to motion artifact due to breathing. Due to the relatively long acquisition times needed for T_1_ mapping, we wanted to limit the potential of motion artifacts interfering with the T_1_ calculation. Therefore, we used a subcutaneous model where the tumor could be placed near the spine to mitigate motion. Methods for T_1_ mapping in clinically feasible times are being approached [50].

In the future, this affords the potential for multiparametric imaging including dynamic contrast imaging to evaluate vascular permeability, diffusion weighted imaging to assess molecular motion, and T_1_ mapping to evaluate T_1_-relaxivity to predict response to anti-angiogenic therapy. Methods for simultaneous quantification of relative proton density, T_1_, and T_2_ in time frames approachable for clinical use [50] suggest the potential of expanded multiparametric quantitative MR, including T_1_ relaxation time measurements, to be applied to patients with ovarian cancer.

### Conclusion

Findings suggest that increased tumor T_1_ relaxation time is associated with response to bevacizumab therapy in ovarian cancer model and might serve as an early indicator of response.

## Supporting Information

S1 Fig Difference in T1-map value day 2 or week 2 vs pre-therapy for individual tumors in treatment groups from Fig 4. (TIF)
