# Supplementary figures and images for: Tumor T1 Relaxation Time for Assessing Response to Bevacizumab Anti-Angiogenic Therapy in a Mouse Ovarian Cancer Model
Source: PLoS One. 2015 Jun 22;10(6):e0131095. doi: 10.1371/journal.pone.0131095 (PMC4476738; doi:10.1371/journal.pone.0131095)

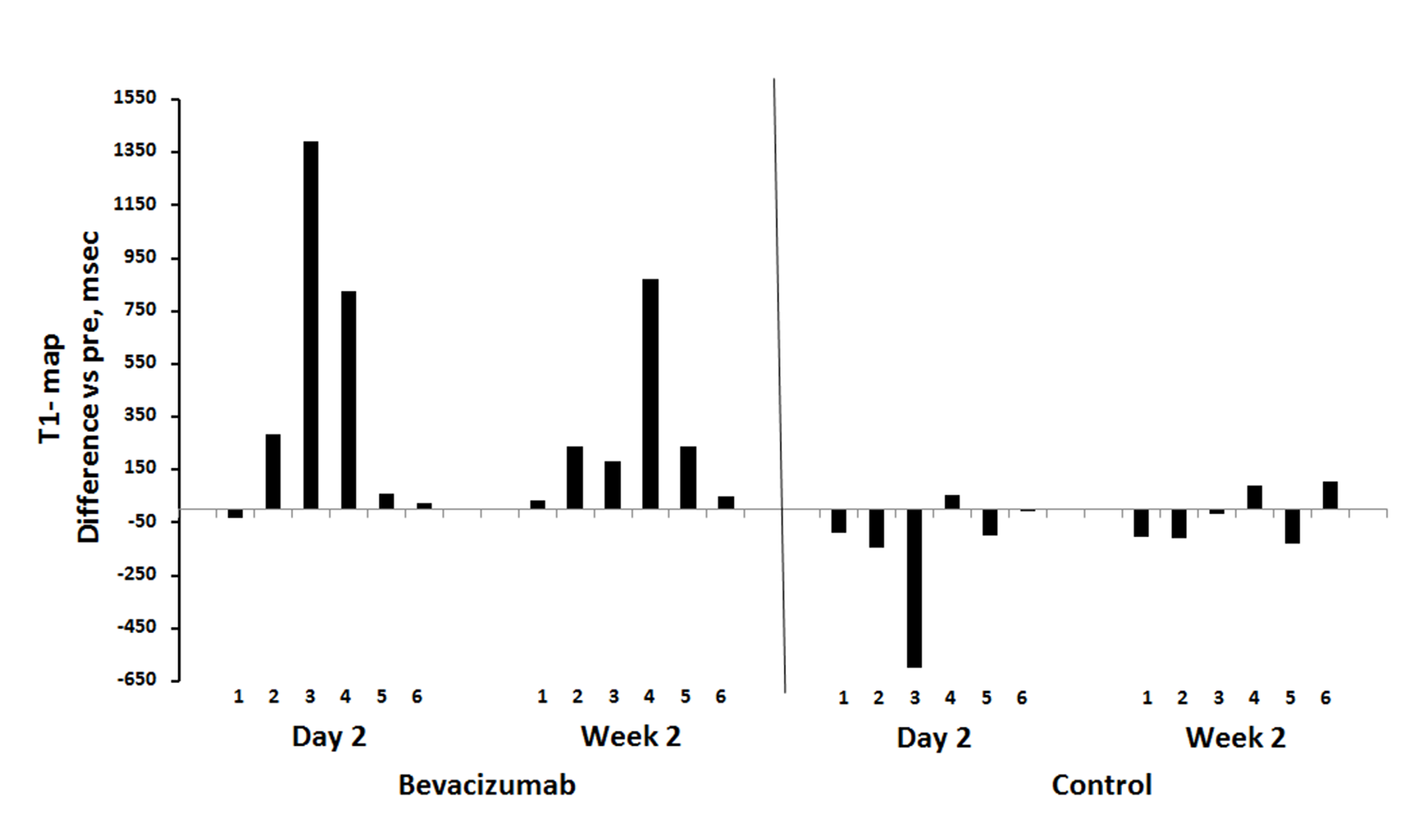

Supplement: S1 Fig — (TIF) [file pone.0131095.s001.tif]
